# Human papillomavirus in semen and the risk for male infertility: a systematic review and meta-analysis

**DOI:** 10.1186/s12879-017-2812-z

**Published:** 2017-11-09

**Authors:** Zhangyan Lyu, Xiaoshuang Feng, Ni Li, Wei Zhao, Luopei Wei, Yuheng Chen, Wenjing Yang, Hongxia Ma, Bing Yao, Kai Zhang, Zhibin Hu, Hongbing Shen, Dong Hang, Min Dai

**Affiliations:** 1 Program Office for Cancer Screening in Urban China, National Cancer Center / Cancer Hospital, Chinese Academy of Medical Sciences and Peking Union Medical College, No.17 Panjiayuannanli, Chaoyang District, Beijing, 100021 China; 2 Reproductive Medical Center, Jinling Hospital, Nanjing, 210029 China; 3 Department of Epidemiology and Biostatistics, Jiangsu Key Lab of Cancer Biomarkers, Prevention and Treatment, Collaborative Innovation Center For Cancer Personalized Medicine, School of Public Health, Nanjing Medical University, Nanjing, 211166 China; 4 State Key Laboratory of Reproductive Medicine, Nanjing Medical University, Nanjing, 211166 China; 5 Department of Cancer Prevention, National Cancer Center / Cancer Hospital, Chinese Academy of Medical Sciences and Peking Union Medical College, Beijing, 100021 China

**Keywords:** Human papillomavirus, Semen, Prevalence, Infertility, Meta-analysis

## Abstract

**Background:**

Human papillomavirus (HPV) is one of the most prevalent sexually transmitted viruses. Despite the increasing evidence of HPV prevalence in semen, the worldwide distribution of HPV types in semen and risk for male infertility remain inconclusive.

**Methods:**

Four electronic databases were searched for English language studies conducted between January 1990 and December 2016 that reported HPV DNA prevalence in semen. Based on the PRISMA guidelines, HPV prevalence was estimated among general population and fertility clinic attendees, respectively, and heterogeneity testing was performed using Cochran’s Q and *I* ^2^ statistics. The association between HPV positivity and male infertility was evaluated by a meta-analysis of case-control studies.

**Results:**

A total of 31 eligible studies comprising 5194 males were included. The overall prevalence of HPV DNA in semen was 11.4% (95% CI = 7.8-15.0%) in general population (*n* = 2122) and 20.4% (95% CI = 16.2-24.6%) in fertility clinic attendees (*n* = 3072). High-risk type prevalence was 10.0% (95% CI = 5.9-14.0%) and 15.5% (95% CI = 11.4-19.7%), respectively. HPV16 was the most common type, with a prevalence of 4.8% (95% CI = 1.7-7.8%) in general population and 6.0% (95% CI = 3.8-8.2%) in fertility clinic attendees. A significantly increased risk of infertility was found for males with HPV positivity in semen (OR = 2.93, 95% CI = 2.03-4.24).

**Conclusions:**

Seminal HPV infection is common worldwide, which may contribute to the risk of male infertility.

**Electronic supplementary material:**

The online version of this article (10.1186/s12879-017-2812-z) contains supplementary material, which is available to authorized users.

## Background

Infertility is defined as the inability of a couple to conceive after 1 year of unprotected sex. Worldwide, approximately 10% to 20% of couples at the reproductive age were affected by infertility. Among them, male factor infertility contributes to roughly 50% of cases, and the proportion is increasing rapidly [1]. Several risk factors have been proposed for male infertility, such as sexual dysfunction, varicocele, congenital dysplasia, endocrine disorders, immune factors, and sexually transmitted infections (STI) [2]. However, in approximately 50% of infertile men, the etiology remains unknown and it is termed idiopathic infertility, only showing oligospermia, asthenospermia, teratozoospermia or other sperm abnormalities.

Recent evidence suggests that HPV infection can be present in semen and is implicated in male infertility [3]. HPV is one of the most common sexually transmitted virus in both males and females worldwide. Over 170 types of HPV have been identified and among those, at least 40 types could infect the anogenital region [4]. A subset of high-risk types (HPV16, 18, etc.) have been proved to cause neoplasms at different sites, such as cervical, vulva, vagina and anus, while low-risk types (HPV6, 11, etc.) mainly result in benign papilloma or no clinical symptoms [5]. In fact, HPV is an organism with two phases, the virion and the infected cell. HPV virions, which are only assembled in non-dividing cells, can induce the infectious virion producing pathway and the clonal transforming pathway [6]. Although the clonal HPV E6/E7 transforming pathway that causes cancers has been well acknowledged, HPV virion related health consequences are greatly underestimated. Actually, HPV DNA measured in semen samples primarily originates from HPV virions and is infectious [6]. HPV virions can lie not only in the perianal region and external genitalia, including the penis foreskin, scrotum and glans penis, but also in the urethra, ductus deferens, epididymis, and testis [7].

Biological evidences indicate that HPV virions could bind two distinct equatorial regions of the sperm head and affect sperm quality, thereby increasing the risk of male infertility [8, 9]. A previous systematic review reported HPV DNA prevalence in semen varying from 0 to 100% [10]. According to a meta-analysis of seven studies focusing on populations seeking fertility evaluation or treatment, the pooled prevalence was 16% (95%CI = 10-23%) [10]. However, the worldwide distribution of seminal HPV types has not been systematically studied, and epidemiologic evidences on the association between seminal HPV infection and infertility are inconsistent. Therefore, we conducted a systematic review and meta-analysis to determine global prevalence and distribution of HPV types in semen. Moreover, we evaluated the association between seminal HPV infection and male infertility.

## Methods

### Literature search

Four electronic databases, including Medline, Embase, ScienceDirect and Cochrane library, were searched systematically for studies on HPV prevalence in semen, with the following MeSH terms: “Papillomaviridae”, “Semen”, “Spermatozoa”, and “Infertility, Male”, published in English during the period from January 1990 to December 2016. Relevant additional references cited in retrieved articles were also evaluated. MOOSE guidelines and the PRISMA statement were followed to conduct the search [11, 12]. The strategy used for searching PubMed is shown in the Additional file 1: Figure S1.

### Eligibility criteria

**Fig. 1:**
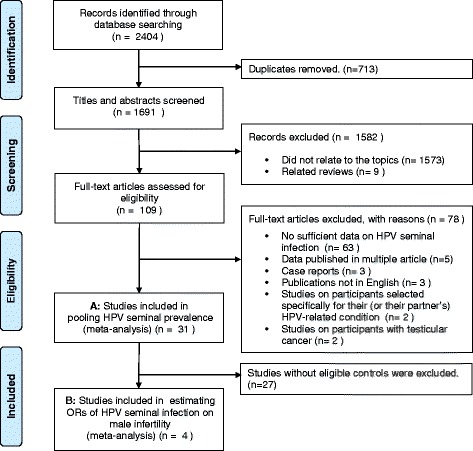
Flow Diagram of the Search Strategy and Exclusion Criteria

Studies were included if HPV DNA prevalence in spermatozoa or whole semen of participants could be directly extracted or calculated from the original article. If data subsets were published in more than one article, only the report with the largest sample size or more detailed information was included. Studies were excluded if they were: 1) reports focusing on participants who never had intercourse, fertility clinic attendees not seeking fertility evaluation, or those with penile warts or testicular cancer; 2) case reports or reviews. The work flow is shown in the Fig. 1.

Male infertility is defined based on the following criteria: at least 1 year of unprotected sexual intercourse without conception, and/or abnormal results of semen analysis referring to the standards in the guidelines of the WHO Laboratory Manual for the Examination and Processing of Human Semen (5th edition) [13].

Study populations were separated into two groups: general population that comprised fertile men and healthy male volunteers, and fertility clinic attendees who were either confirmed infertile men or seeking ertility evaluation and assisted reproductive techniques (ART). Participants diagnosed with infertility in general population and fertile men identified in fertility clinic attendees were excluded. These two groups were created to represent low-risk and high-risk infertility populations, respectively. Only case-control studies including infertile men as cases and confirmed fertile men as controls were utilized for the meta-analysis of association between HPV positivity and male infertility.

### Data extraction

The data of included articles were extracted independently by two reviewers (ZL and XF). Discrepancies were discussed and resolved by consensus (NL, DH and MD). For each study included, key extracted information included: first author, publication year, study design, population characteristics (country of region, age, sample size, general population or fertility clinic attendees), method of semen preservation (fresh or frozen), HPV detection method [polymerase chain reaction (PCR) based or not], types of primers, HPV overall and type-specific prevalence, and matching criteria if controls were present. Detailed information on all included studies is presented in Additional file 2: Table S1.

### Statistical analysis

HPV prevalence was computed by dividing the number of HPV DNA positive cases by the total number of cases tested. We calculated overall and type-specific prevalence in semen, as well as corresponding two-sided 95% confidence intervals (CIs). HPV16, 18, 31, 33, 35, 39, 45, 51, 52, 56, 58, 59 and 68 were grouped as high-risk types, and HPV6, 11, 40, 42, 43, 53, 54, 56, 61, 62, 66 and 70 were grouped as low-risk types. Stratified analyses were performed with respect to potential influential parameters, and the difference in prevalence between the strata was assessed by χ^2^ tests. Furthermore, the association between seminal HPV infection and male infertility risk was estimated by odds ratio (OR) with 95% CIs in logistic regression models.

Inter-study heterogeneity was assessed with Cochran’s χ^2^-based Q test, and the percentage of total variation across studies was evaluated with the *I* ^*2*^ measure. When inter-study heterogeneity was non-significant, a fixed-effects model based on the Mantel-Haenszel method was used to pool the data; otherwise, a random-effects model based on the DerSimonian and Laird method was chosen. Sensitivity analysis was conducted to assess the influence of individual study on the strength and stability of the meta-analytic results through omitting one study at a time. Publication bias was evaluated using Egger’s linear regression test [14] and Begg’s rank correlation test [15]. All statistical tests were two-sided and statistical significance was defined as *P* value less than 0.05. Statistical analyses were performed using STATA (version 12.0) and SAS (version 9.4).

## Results

### Study characteristics

To estimate worldwide distribution of seminal HPV infection, a total of 31 eligible studies were identified, including 11 studies [8, 16–25] reporting HPV prevalence in general population, 16 studies [26–41] in fertility clinic attendees, and 4 studies [42–45] in both populations. In most studies, the HPV detection was based on PCR method with consensus or type-specific primers. Finally, 738 HPV DNA positive men among 5194 participants from 16 countries were pooled into the meta-analysis.

In 15 studies on the HPV prevalence of general population, 6 studies included a total of 815 confirmed fertile males and the other 9 studies included 1307 healthy male volunteers. In 20 studies on the HPV prevalence of fertility clinic attendees, 5 studies included 1002 confirmed infertile men and the other 15 studies included 2070 male partners of infertile couples who sought fertility evaluation or ART.

### HPV prevalence in semen

The reported HPV DNA prevalence in semen ranged from 0.0% to 46.2% (Additional file 2: Table S1) and yielded an average of 17.1% (95% CI = 14.1-20.1%). The pooled prevalence in fertility clinic attendees (20.4%, 95% CI = 16.2-24.6%) was significantly higher than that in general population (11.4%, 95% CI = 7.8-15.0%) (*P* < 0.001). High-risk HPV prevalence (10.0%, 95% CI = 5.9-14.0%) was similar to that of low-risk types (8.3%, 95% CI = 4.1-12.5%) in general population (*P* = 0.072). However, in fertility clinic attendees, the prevalence of high-risk HPV (15.5%, 95% CI = 11.4-19.7%) was significantly higher than that of low-risk types (10.3%, 95% CI = 6.8-13.9%) (*P* < 0.001).

A total of 24 HPV types (HPV6, 11, 16, 18, 31, 33, 35, 39, 40, 42, 45, 51, 52, 53, 54, 56, 58, 59, 61, 62, 66, 68, 70 and 81) were detected in semen samples across studies (Table 1). Two most common high-risk types in general population and fertility clinic attendees were HPV16 (4.8%, 95% CI = 1.7-7.8%; 6.0%, 95% CI = 3.8-8.2%, respectively) and HPV56 (3.8%, 95% CI = 1.2-6.3%; 1.9%, 95% CI = 0.0-3.9%, respectively). The most common low-risk type was HPV6, with the prevalence of 2.4% (95% CI = 0.3-4.5%) in general population and 1.3% (95% CI = 0.3-2.3%) in fertility clinic attendees (Table 1).

**Table 1:** Seminal HPV prevalence by types according to population

	General Population			Fertility Clinic Attendees		
	No. of studies	No. of HPV positive	HPV DNA prevalence (%) (95%CI^a^)	No. of studies	No. of HPV positive	HPV DNA prevalence(%) (95%CI^a^)
Total	15	221	11.4 (7.8-15.0)	20	517	20.4 (16.2-24.6)
High-risk	10	123	10.0 (5.9-14.0)	15	314	15.5 (11.4-19.7)
Low-risk	7	97	8.3 (4.1-12.5)	10	166	10.3 (6.8-13.9)
Individual Type						
Clade 9	7	67	7.4 (3.3-11.6)	14	197	10.6 (7.3-13.9)
HPV16	7	43	4.8 (1.7-7.8)	14	122	6.0 (3.8-8.2)
HPV31	3	9	1.1 (0.0-2.5)	5	17	1.0 (0.5-1.5)
HPV33	2	5	0.8 (0.1-1.5)	4	12	0.6 (0.0-1.2)
HPV35	1	2	1.1 (0.4-2.5)	2	3	0.4 (0.0-0.8)
HPV52	2	6	1.2 (0.0-3.5)	7	27	1.2 (0.6-1.7)
HPV58	2	2	0.6 (0.0-1.6)	4	16	0.8 (0.0-2.1)
Clade 7	6	31	2.3(1.3-3.3)	8	93	4.1 (1.7-6.5)
HPV18	6	11	0.8 (0.1-1.6)	8	33	1.1 (0.1-2.1)
HPV39	1	3	0.6 (0.0-1.2)	3	9	0.5 (0.0-1.0)
HPV45	1	1	0.2 (0.0-0.6)	4	22	1.2 (0.1-2.3)
HPV59	3	7	0.9 (0.0-2.1)	4	19	1.1 (0.3-1.9)
HPV68	1	7	1.3 (0.4-2.3)	2	6	0.5 (0.1-1.0)
HPV70	1	2	0.2 (0.0-4.7)	4	4	0.2 (0.0-0.5)
Clade 10	5	18	2.6 (0.6-4.6)	10	60	2.4 (1.0-3.7)
HPV6	5	15	2.4 (0.3-4.5)	6	26	1.3 (0.3-2.3)
HPV11	2	3	0.4 (0.0-0.9)	4	29	1.9 (0.0-3.8)
HPV44	0	–	–	4	5	0.8 (0.0-1.6)
Clade 3	3	14	1.6 (0.7-2.4)	6	15	0.5 (0.1-1.0)
HPV61	2	3	1.0 (0.0-2.2)	2	2	0.3 (0.0-0.8)
HPV62	2	3	1.0 (0.0-2.2)	3	8	1.7 (0.5-2.9)
HPV81	2	8	1.0 (0.2-1.7)	3	5	0.4 (0.0-0.7)
Clade 6	6	29	2.9 (0.6-5.2)	9	61	2.8 (1.4-4.2)
HPV53	4	6	0.4 (0.0-1.0)	5	17	1.0 (0.2-1.7)
HPV56	1	8	3.8 (1.2-6.3)	4	18	1.9 (0.0-3.9)
HPV66	5	15	1.8 (0.2-3.4)	9	26	0.9 (0.5-1.4)
Clade 8	1	1	0.2 (0.0-0.6)	5	15	1.0 (0.1-1.8)
HPV40	1	1	0.2 (0.0-0.6)	3	5	0.5 (0.0-1.4)
HPV43	0	–	–	3	10	0.7 (0.0-1.9)
Clade 1						
HPV42	3	15	3.1 (0.0-7.7)	4	20	1.2 (0.5-1.9)
Clade 5						
HPV51	3	16	2.7 (0.7-4.6)	7	16	0.7 (0.2-1.3)
Clade 13						
HPV54	2	3	0.4 (0.0-0.9)	6	14	0.7 (0.1-1.3)

^**a**^ *Abbreviations*: *CI* confidence interval

### Stratified analysis

In the stratified analysis by study region, overall prevalence of HPV DNA in semen among general population was the highest in Europe (15.2%, 95% CI = 8.3-22.1%), followed by Asia (9.2%, 95% CI = 0.3-18.0%) (*P* < 0.001) and North America (4.5%, 95% CI = 2.9-6.0%) (*P* < 0.001). For fertility clinic attendees, the overall prevalence was the highest in Latin America (38.2%, 95% CI = 27.2-49.1%), followed by Ocean (29.4%, 95% CI = 7.8-51.1%) (*P* = 0.498), Africa (28.6%, 95% CI = 17.4-39.7%) (*P* = 0.234), Asia (22.0%, 95% CI = 11.4-32.5%) (*P* < 0.001), North America (19.3%, 95% CI = 0.0-49.8%) (*P* < 0.001) and Europe (17.9%, 95% CI = 13.0-22.7%) (*P* = 0.001).

Semen specimens were either used directly or centrifuged to collect sperms for subsequent HPV analysis. Among general population, HPV prevalence in non-centrifuged semen (12.4%, 95% CI = 6.7-18.0%) was similar to that in centrifuged semen (10.9%, 95% CI = 5.4-16.3%, *P* = 0.346). For fertility clinic attendees, HPV prevalence was also comparable in non-centrifuged semen (20.8%, 95% CI = 13.1-28.5%) and centrifuged semen (18.0%, 95% CI = 12.8-23.3%; *P* = 0.201).

As to HPV detection techniques, PCR-based methods with consensus spectrum primers or type-specific primers, and non-PCR methods including in-situ hybridization (ISH) were commonly employed. In general population, HPV prevalence was 19.0% (95% CI = 5.2-32.8%) when type-specific PCR primers were used, and it was 11.0% (95% CI = 7.3-14.7%) when consensus primers were used (*P* = 0.128). The analysis in fertility clinic attendees also showed relatively higher prevalence when using type-specific primers than using consensus primers (27.4%, 95% CI = 8.5-46.2%; 18.7%, 95% CI = 14.2-23.1%, respectively; *P* = 0.002) (Table 2).

**Table 2:** Seminal HPV prevalence by region, specimen type, and detection method

	General Population			Fertility Clinic Attendees		
	No. of studies	No. of HPV positive	HPV DNA prevalence (%) (95%CI^a^)	No. of studies	No. of HPV positive	HPV DNA prevalence (%) (95%CI^a^)
Region						
Europe	10	149	15.2 (8.3-22.1)	10	285	17.9 (13.0-22.7)
Asia	2	39	9.2 (0.3-18.0)	5	157	22.0 (11.4-32.5)
North America	3	33	4.5 (2.9-6.0)	2	23	19.3 (0.0-49.8)
Africa	0	–	–	1	18	28.6 (17.4-39.7)
Ocean	0	–	–	1	5	29.4 (7.8-51.1)
Latin America	0	–	–	1	29	38.2 (27.2-49.1)
Specimen type						
Non-centrifuged semen	9	133	12.4 (6.7-18.0)	7	205	20.8 (13.1-28.5)
Centrifuged semen	6	88	10.9 (5.4-16.3)	11	278	18.0 (12.8-23.3)
Unclear	0	–	–	2	34	36.4 (26.6-46.1)
Detection method						
Consensus primers	13	215	11.0 (7.3-14.7)	14	397	18.7 (14.2-23.1)
Type-specific primers	2	6	19.0 (5.2-32.8)	5	78	27.4 (8.5-46.2)
Others	0	–	–	1	42	18.6 (13.5-23.7)

^**a**^ *Abbreviations*: *CI* confidence interval

### Association between HPV positivity in semen and male infertility

**Fig. 2:**
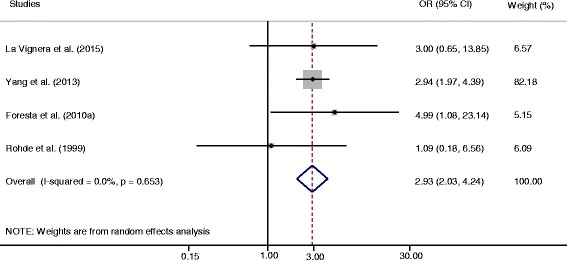
Forest plots with odds ratios (ORs) of male infertility for HPV seminal infection

To assess the association between seminal HPV infection and male infertility risk, four eligible case-control studies including 853 infertile patients and 641 fertile controls were analyzed [42–45]. According to the results of the heterogeneity test (*Q* = 1.63, *P* = 0.653, *I* ^*2*^ = 0.00%), the fixed-effects model was chosen to estimate the pooled OR. We found that HPV positivity was significantly associated with an increased risk of infertility (OR = 2.93, 95% CI = 2.03-4.24) (Fig. 2).

The sensitivity analysis showed that OR estimates ranged from 2.82 (95% CI: 1.93-4.12) to 3.05 (95% CI: 2.10-4.45), suggesting that no single study influenced the stability of the association (Additional file 3: Figure S2). Egger’s and Begg’s tests indicated no significant publication bias (*P* = 0.808 and 0.734, respectively).

## Discussion

The current study provides an important overview on HPV DNA prevalence in semen and its relationship with male infertility. The results showed that the pooled HPV prevalence was significantly higher in fertility clinic attendees (20.4%, 95% CI = 16.2-24.6%) than in general population (11.4%, 95% CI = 7.8-15.0%). Moreover, a meta-analysis of case-control studies revealed a significant association between seminal HPV infection and male infertility (OR = 2.93, 95% CI = 2.03-4.24).

According to the meta-analysis by Laprise et al. in 2014, the pooled prevalence of seven studies focusing on populations seeking fertility evaluation or treatment was 16% (95% CI = 10-23%) [10],lower than 20.4% (95% CI = 16.2-24.6%) in our present study. We included six additional studies published after 2014 for fertility clinic attendees, of which four studies with relatively large sample size reported HPV prevalence higher than 16% [27, 28, 30, 42]. In addition, Laprise et al. excluded the studies that targeted less than 20 HPV types, but we made no such exclusion and analyzed type-specific HPV prevalence in semen.

In terms of HPV type distribution, mounting evidence have shown that high-risk HPV16 was predominant in male anogenital sites, prostate, bladder, and oropharynx [46, 47]. Our study indicated that HPV16 was the most common type in semen, accounting for approximately one fifth of HPV-positive samples from both general population and fertility clinic attendees. We also found that the prevalence of high-risk HPV56, which is less commonly detected in HPV-related cancers, ranked second after HPV16 in semen. In fact, clonal HPV (not infectious) is mainly responsible for cancer initiation, and infectious HPV virions could contribute to subfertility [6]. HPV virions are only assembled in non-dividing cells, and sperm and seminal plasma does not normally contain dividing cells. Thus, HPV DNA measured in semen mostly originates from virions [6]. Considering that HPV virions are mainly transmitted through sexual behaviors and HPV56 belongs to non-vaccine types, its presence in semen should be given more attention.

To the best of our knowledge, the current study was the first to examine the geographical variation of seminal HPV prevalence, showing a relatively high prevalence in fertility clinic attendees in Latin America and Africa. A previous meta-analysis of cervical HPV infection in women with normal cytology has reported a relatively high prevalence in Africa and Latin America [48]. Since HPV virions can be sexually transmitted, it is reasonable that seminal HPV prevalence exhibits a similar geographical variation to cervical HPV infection.

Type-specific PCR method was commonly used to detect HPV DNA before consensus PCR which has been widely applied since 2000. However, evidence shows that the sensitivity and specificity of consensus PCR were lower than those of type-specific PCR [49]. In addition, type-specific PCR targeting a specific region in the HPV genome (e.g. E6/E7) seems more suitable in cervical screening [50]. The present study found that seminal HPV prevalence was higher in studies using type-specific primers than in those using consensus primers. Thus, type-specific PCR may represent a better choice to detect HPV in semen, especially when HPV DNA copy number is low in semen samples.

Studies without proper control group are unable to determine the effect of HPV infection on male infertility [6]. So far, only a limited number of case-control studies have been conducted and their results are inconsistent. For example, a case-control study conducted in China showed that infertile males had significantly higher HPV prevalence in semen (17.4%) than fertile controls (6.7%) [43]; however, another study failed to confirm the association [45]. The inconsistency may be partly attributed to the relatively small sample size of individual studies. Therefore, we conducted a meta-analysis of published case-control studies to evaluate the association and its strength between HPV positivity and male infertility. Our findings suggested that men with seminal HPV infection had a two-fold increased risk of infertility compared with those without infection. To assess potential bias due to the quality of included studies, the sensitivity analysis was conducted by calculating pooled OR when omitting each one study, and the results were robust.

Several pathogenic mechanisms have been proposed to explain the effect of seminal HPV infection on male infertility. Firstly, HPV virions may lead to a significant impairment of sperm parameters (e.g. concentration, morphology, and pH), especially a reduction in sperm motility, thereby affecting male fertility [51]. Lai et al. first reported the lower performance of curvilinear velocity, straight-line velocity and mean amplitude of lateral head displacement in HPV-infected sperm [38], and the finding was confirmed in the other studies [18, 25, 43, 44]. Secondly, HPV infection is considered a risk factor for anti-sperm antibodies (ASAs) which may reduce male fertility by interfering with sperm motility and sperm-oocyte binding, and by mediating the release of cytokines that can impair sperm function. Garolla et al. demonstrated the presence of HPV DNA at sperm level is frequently associated with ASAs of IgA and IgG classes in infertile patients [52]. Thirdly, the ability of sperms to carry exogenous HPV into oocytes and viral genome into the blastocysts and its impact on fertility have been demonstrated. Lai et al. first suggested that spermatozoa can act like a vector for HPV transmission to sexual partners and to fetus through fertilized eggs [53]. The role of HPV infection in adverse pregnancy outcomes (e.g. miscarriage) has been validated by subsequent studies [3]. Finally, HPV infection might affect the integrity of sperm DNA. Connelly et al. reported that sperm cells transfected with exogenous HPV E6/E7 DNA had higher percentages of breakages characteristic of apoptosis compared to the uninfected controls [54]. However, in vivo study by Cortes et al. failed to observe the increased DNA fragmentation in semen containing HPV [55]. Future studies with large sample size and rigorous design are necessary to confirm whether HPV-positive spermatozoa are more susceptible to DNA damage.

Several limitations of the present study should be considered when interpreting the results. Firstly, though we conducted a comprehensive literature research, potential selection bias could not be completely excluded because only articles published in English were included. Secondly, it is possible that the analysis of our low-risk infertility population overestimated HPV prevalence for fertile males, and the high-risk infertility population underestimated the prevalence for infertile males, introducing a bias toward null assumption. However, the pooled prevalence in the high-risk population was still significantly higher than that in the low-risk population, supporting the role of HPV infection in male infertility. Thirdly, there was potential heterogeneity between studies different in study design, sample size, ethnicity, and number of HPV types detected. Examining possible causes of heterogeneity by the stratified analysis yielded little additional insight into sources of heterogeneity. Lastly, only four case-control studies were included to evaluate the association between seminal HPV positivity and male infertility. Additional studies with large sample size are needed to verify our current findings.

## Conclusions

The current study suggests that seminal HPV infection is prevalent worldwide, which may affect male fertility. Prospective cohort studies and functional experiments are needed to increase our knowledge on the implication of HPV infection in reproductive health.

## Additional files

Additional file 1: Figure S1. The full search strategy used for searching PubMed. This figure should be placed after the line 120. (PDF 118 kb)

Additional file 2: Table S1. Studies Included in the Meta-analysis and Their Characteristics by Region. This table should be placed after the line 152. (XLSX 33 kb)

Additional file 3: Figure S2. Sensitivity Analysis for Individual Studies on the Summary Effect. This figure should be placed after the line 247. (PDF 98 kb)

## Abbreviations

ART: Assisted reproductive techniques
ASAs: Anti-sperm antibodies
CIs: Confidence intervals
HPV: Human papillomavirus
ISH: In-situ hybridization
OR: Odds ratio
PCR: Polymerase chain reaction
STI: Sexually transmitted infections

## References

[1] Agarwal A, Mulgund A, Hamada A, Chyatte MR (2015). A unique view on male infertility around the globe. Reprod Biol Endocrinol.

[2] Krausz C (2011). Male infertility: pathogenesis and clinical diagnosis. Best Pract Res Clin Endocrinol Metab.

[3] Gizzo S, Ferrari B, Noventa M, Ferrari E, Patrelli TS, Gangemi M, Nardelli GB (2014). Male and couple fertility impairment due to HPV-DNA sperm infection: update on molecular mechanism and clinical impact--systematic review. Biomed Res Int.

[4] de Villiers EM (2013). Cross-roads in the classification of papillomaviruses. Virology.

[5] Bouvard V, Baan R, Straif K, Grosse Y, Secretan B, Ghissassi FE, Benbrahim-Tallaa L, Guha N, Freeman C, Galichet L (2009). A review of human carcinogens—part B: biological agents. Lancet Oncol.

[6] Depuydt CE, Beert J, Bosmans E, Salembier G (2016). Human Papillomavirus (HPV) virion induced cancer and subfertility, two sides of the same coin. Facts, views & vision in ObGyn.

[7] Dunne EF, Nielson CM, Stone KM, Markowitz LE, Giuliano AR (2006). Prevalence of HPV infection among men: a systematic review of the literature. J Infect Dis.

[8] Kaspersen MD, Larsen PB, Ingerslev HJ, Fedder J, Petersen GB, Bonde J, Höllsberg P. Identification of multiple HPV types on spermatozoa from human sperm donors. PLoS One. 2011;6(3) doi:10.1371/journal.pone.0018095. 10.1371/journal.pone.0018095 PMC3066218 21479232

[9] Perez-Andino J, Buck CB, Ribbeck K (2009). Adsorption of human papillomavirus 16 to live human sperm. PLoS One.

[10] Laprise C, Trottier H, Monnier P, Coutlee F, Mayrand MH: Prevalence of human papillomaviruses in semen: a systematic review and meta-analysis. Human reproduction (Oxford, England) 2014, 29(4):640–651. 10.1093/humrep/det453 24365799

[11] Stroup DF, Berlin JA, Morton SC, Olkin I, Williamson GD, Rennie D, Moher D, Becker BJ, Sipe TA, Thacker SB (2000). Meta-analysis of observational studies in epidemiology: a proposal for reporting. Meta-analysis of observational studies in epidemiology (MOOSE) group. JAMA.

[12] Moher D, Clarke M, Ghersi D, Liberati A, Petticrew M, Shekelle P, Stewart LA, Shamseer L (2015). Group P-P: preferred reporting items for systematic review and meta-analysis protocols (PRISMA-P) 2015: elaboration and explanation. BMJ (Clinical research ed).

[13] World Health Organization (2010). WHO laboratory manual for the examination and processing of human semen Fifth edition. Cambridge, UK: Cambridge University Press. [http://www.who.int/reproductivehealth/publications/infertility/9789241547789/en/]. Accessed 4 July 2017.

[14] Egger M, Davey Smith G, Schneider M, Minder C (1997). Bias in meta-analysis detected by a simple, graphical test. BMJ (Clinical research ed).

[15] Begg CB, Mazumdar M (1994). Operating characteristics of a rank correlation test for publication bias. Biometrics.

[16] Luttmer R, Dijkstra MG, Snijders PJF, Jordanova ES, King AJ, Pronk DTM, Foresta C, Garolla A, Hompes PGA, Berkhof J (2015). Presence of human papillomavirus in semen of healthy men is firmly associated with HPV infections of the penile epithelium. Fertil Steril.

[17] Kero K, Rautava J, Syrjänen K, Grenman S, Syrjänen S (2011). Human Papillomavirus genotypes in male genitalia and their concordance among pregnant spouses participating in the Finnish family HPV study. J Sex Med.

[18] Foresta C, Garolla A, Zuccarello D, Pizzol D, Moretti A, Barzon L, Palù G (2010). Human papillomavirus found in sperm head of young adult males affects the progressive motility. Fertil Steril.

[19] Hernandez BY, Wilkens LR, Zhu X, McDuffie K, Thompson P, Shvetsov YB, Ning L, Goodman MT (2008). Circumcision and human papillomavirus infection in men: a site-specific comparison. J Infect Dis.

[20] Giuliano AR, Nielson CM, Flores R, Dunne EF, Abrahamsen M, Papenfuss MR, Markowitz LE, Smith D, Harris RB (2007). The optimal anatomic sites for sampling heterosexual men for human papillomavirus (HPV) detection: the HPV detection in men study. J Infect Dis.

[21] Rintala MAM, Grénman SE, Pöllänen PP, Suominen JJO, Syrjänen SM (2004). Detection of high-risk HPV DNA in semen and its association with the quality of semen. Int J STD AIDS.

[22] Rintala MA, Pollanen PP, Nikkanen VP, Grenman SE, Syrjanen SM (2002). Human papillomavirus DNA is found in the vas deferens. J Infect Dis.

[23] Olatunbosun O, Deneer H, Pierson R (2001). Human papillomavirus DNA detection in sperm using polymerase chain reaction. Obstet Gynecol.

[24] Inoue M, Nakazawa A, Fujita M, Tanizawa O (1992). Human papillomavirus (HPV) type 16 in semen of partners of women with HPV infection. Lancet.

[25] Garolla A, Lenzi A, Palu G, Pizzol D, Bertoldo A, De Toni L, Foresta C (2012). Human papillomavirus sperm infection and assisted reproduction: a dangerous hazard with a possible safe solution. Human reproduction (Oxford England).

[26] Luttmer R, Dijkstra MG, Snijders PJ, Hompes PG, Pronk DT, Hubeek I, Berkhof J, Heideman DA, Meijer CJ (2016). Presence of human papillomavirus in semen in relation to semen quality. Human reproduction (Oxford, England).

[27] Garolla A, Engl B, Pizzol D, Ghezzi M, Bertoldo A, Bottacin A, Noventa M, Foresta C (2016). Spontaneous fertility and in vitro fertilization outcome: new evidence of human papillomavirus sperm infection. Fertil Steril.

[28] Nasseri S, Monavari SH, Keyvani H, Nikkhoo B, Vahabpour Roudsari R, Khazeni M (2015). The prevalence of human Papilloma virus (HPV) infection in the oligospermic and azoospermic men. Med J Islam Repub Iran.

[29] Golob B, Poljak M, Verdenik I, Kolbezen Simoniti M, Vrtačnik Bokal E, Zorn B. High HPV infection prevalence in men from infertile couples and lack of relationship between seminal HPV infection and sperm quality. Biomed Res Int. 2014; doi:10.1155/2014/956901. 10.1155/2014/956901 PMC3997886 24809062

[30] Gimenes F, Medina FS, Abreu AL, Irie MM, Esquicati IB, Malagutti N, Vasconcellos VR, Discacciati MG, Bonini MG, Maria-Engler SS (2014). Sensitive simultaneous detection of seven sexually transmitted agents in semen by multiplex-PCR and of HPV by single PCR. PLoS One.

[31] Schillaci R, Capra G, Bellavia C, Ruvolo G, Scazzone C, Venezia R, Perino A (2013). Detection of oncogenic human papillomavirus genotypes on spermatozoa from male partners of infertile couples. Fertil Steril.

[32] Reich O, Auner H, Puerstner P (2012). Should human papillomavirus testing be performed in men participating in protocols of assisted reproduction?. Int J Androl.

[33] Perino A, Giovannelli L, Schillaci R, Ruvolo G, Fiorentino FP, Alimondi P, Cefalù E, Ammatuna P (2011). Human papillomavirus infection in couples undergoing in vitro fertilization procedures: impact on reproductive outcomes. Fertil Steril.

[34] Didelot-Rousseau MN, Diafouka F, Yayo E, Kouadio LP, Monnet D, Segondy M (2007). HPV seminal shedding among men seeking fertility evaluation in Abidjan, Ivory Coast. J Clin Virol.

[35] Bezold G, Politch JA, Kiviat NB, Kuypers JM, Wolff H, Anderson DJ (2007). Prevalence of sexually transmissible pathogens in semen from asymptomatic male infertility patients with and without leukocytospermia. Fertil Steril.

[36] Czegledy J, Szarka K (2006). Detection of high-risk HPV DNA in semen and its association with the quality of semen. Int J STD AIDS.

[37] Tanaka H, Karube A, Kodama H, Fukuda J, Tanaka T (2000). Mass screening for human papillomavirus type 16 infection in infertile couples. The Journal of reproductive medicine.

[38] Lai YM, Soong YK, Lee JF, Yang FP, Huang HY, Pao CC (1997). The effect of human papillomavirus infection on sperm cell motility. Fertil Steril.

[39] Kyo S, Inoue M, Koyama M, Fujita M, Tanizawa O, Hakura A (1994). Detection of high-risk human papillomavirus in the cervix and semen of sex partners. J Infect Dis.

[40] Chan PJ, BC S, Kalugdan T, Seraj IM, Tredway DR, King A (1994). Human papillomavirus gene sequences in washed human sperm deoxyribonucleic acid. Fertil Steril.

[41] Green J, Monteiro E, Bolton VN, Sanders P, Gibson PE (1991). Detection of human papillomavirus DNA by PCR in semen from patients with and without penile warts. Genitourin Med.

[42] La Vignera S, Vicari E, Condorelli RA, Franchina C, Scalia G, Morgia G, Perino A, Schillaci R, Calogero AE (2015). Prevalence of human papilloma virus infection in patients with male accessory gland infection. Reprod BioMed Online.

[43] Yang Y, Jia CW, Ma YM, Zhou LY, Wang SY (2013). Correlation between HPV sperm infection and male infertility. Asian journal of andrology.

[44] Foresta C, Pizzol D, Moretti A, Barzon L, Palù G, Garolla A (2010). Clinical and prognostic significance of human papillomavirus DNA in the sperm or exfoliated cells of infertile patients and subjects with risk factors. Fertil Steril.

[45] Rohde V, Erles K, Sattler HP, Derouet H, Wullich B, Schlehofer JR (1999). Detection of adeno-associated virus in human semen: does viral infection play a role in the pathogenesis of male infertility?. Fertil Steril.

[46] Ndiaye C, Mena M, Alemany L, Arbyn M, Castellsague X, Laporte L, Bosch FX, de Sanjose S, Trottier H (2014). HPV DNA, E6/E7 mRNA, and p16INK4a detection in head and neck cancers: a systematic review and meta-analysis. The Lancet Oncology.

[47] Yang L, Xie S, Feng X, Chen Y, Zheng T, Dai M, Zhou CK, Hu Z, Li N, Hang D (2015). Worldwide prevalence of human Papillomavirus and relative risk of prostate cancer: a meta-analysis. Sci Rep.

[48] de Sanjose S, Diaz M, Castellsague X, Clifford G, Bruni L, Munoz N, Bosch FX (2007). Worldwide prevalence and genotype distribution of cervical human papillomavirus DNA in women with normal cytology: a meta-analysis. Lancet Infect Dis.

[49] Depuydt CE, Boulet GA, Horvath CA, Benoy IH, Vereecken AJ, Bogers JJ (2007). Comparison of MY09/11 consensus PCR and type-specific PCRs in the detection of oncogenic HPV types. J Cell Mol Med.

[50] Tjalma WA, Depuydt CE (2013). Cervical cancer screening: which HPV test should be used--L1 or E6/E7?. Eur J Obstet Gynecol Reprod Biol.

[51] Foresta C, Noventa M, De Toni L, Gizzo S, Garolla A (2015). HPV-DNA sperm infection and infertility: from a systematic literature review to a possible clinical management proposal. Andrology.

[52] Garolla A, Pizzol D, Bertoldo A, De Toni L, Barzon L, Foresta C (2013). Association, prevalence, and clearance of human papillomavirus and antisperm antibodies in infected semen samples from infertile patients. Fertil Steril.

[53] Lai YM, Yang FP, Pao CC (1996). Human papillomavirus deoxyribonucleic acid and ribonucleic acid in seminal plasma and sperm cells. Fertil Steril.

[54] Connelly DA, Chan PJ, Patton WC, King A (2001). Human sperm deoxyribonucleic acid fragmentation by specific types of papillomavirus. Am J Obstet Gynecol.

[55] Cortes-Gutierrez EI, Davila-Rodriguez MI, Fernandez JL, de la OPLO, Garza-Flores ME, Eguren-Garza R, Gosalvez J. The presence of human papillomavirus in semen does not affect the integrity of sperm DNA. Andrologia. 2017; doi:10.1111/and.12774. 10.1111/and.12774 28261849

