# Supplementary material for: Human papillomavirus in semen and the risk for male infertility: a systematic review and meta-analysis
Source: BMC Infect Dis. 2017 Nov 9;17:714. doi: 10.1186/s12879-017-2812-z (PMC5679371; doi:10.1186/s12879-017-2812-z)
Supplement: Supplementary file 1 — The full search strategy used for searching PubMed. This figure should be placed after the line 120. (PDF 118 kb) [file 12879_2017_2812_MOESM1_ESM.pdf]

(((“Papillomaviridae”[Mesh]) OR “HPV”[Title/Abstract]) OR “Human papillomavirus”[Title/Abstract]) AND ((((((“Semen”[Mesh]) OR “Spermatozoa”[Mesh]) OR “Semen Analysis”[Mesh]) OR “seminal”[Title/Abstract]) OR “Sperm”[Title/Abstract]) OR ((“Infertility, Male”[Mesh] ) OR (“infertility”[Title/Abstract] AND “ men”[Title/Abstract])))
