# Supplementary figures and images for: Human papillomavirus in semen and the risk for male infertility: a systematic review and meta-analysis
Source: BMC Infect Dis. 2017 Nov 9;17:714. doi: 10.1186/s12879-017-2812-z (PMC5679371; doi:10.1186/s12879-017-2812-z)

Meta-analysis estimates, given named study is omitted

| Lower CI Limit    ○ Estimate    | Upper CI Limit

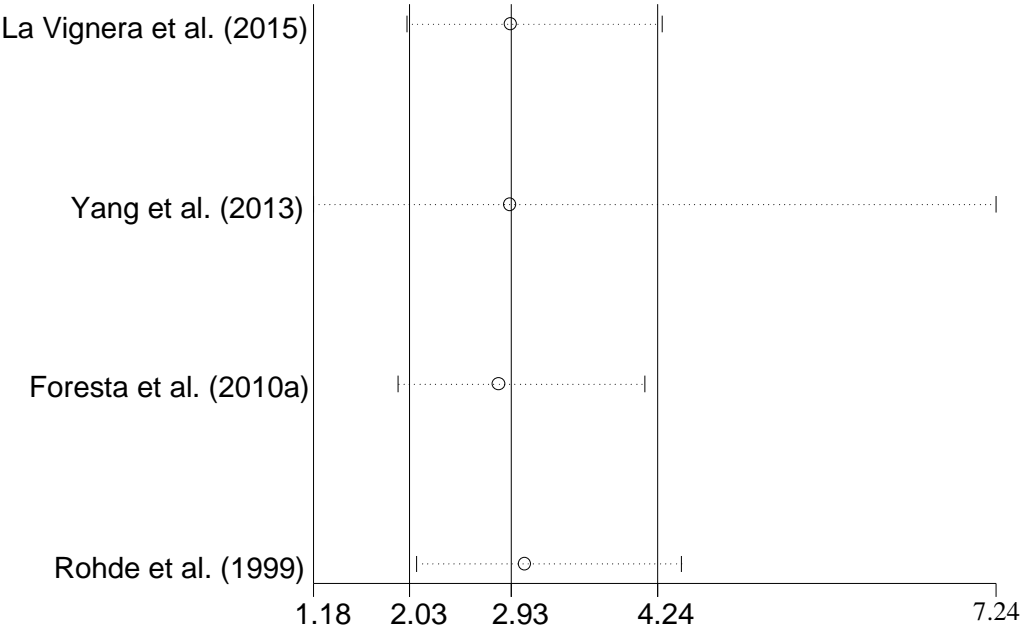

Supplement: Supplementary file 3 — Sensitivity Analysis for Individual Studies on the Summary Effect. This figure should be placed after the line 247. (PDF 98 kb) [file 12879_2017_2812_MOESM3_ESM.pdf]
